# Supplementary material for: Carbon Monoxide Production during Bio-Waste Composting under Different Temperature and Aeration Regimes
Source: Materials (Basel). 2023 Jun 23;16(13):4551. doi: 10.3390/ma16134551 (PMC10342461; doi:10.3390/ma16134551)
Supplement: Supplementary file 1 [file materials-16-04551-s001.zip › Supplementary materials/Table S1.pdf]

Table S1. Properties of composts from different temperature variants ± st. dev.; d.m. – dry matter

Process T, °C	Aeration rate, $L \cdot h^{-1}$	pH	EC, $mS \cdot cm^{-1}$	TOC, % d.m.	TN, % d.m.	C/N	Water content, %	LOI, % d.m.	NH_4-N, mg·kg d.m.$^{-1}$	NO_3-N, mg·kg d.m. $^{-1}$	AT_4, mg O_2·g d.m. $^{-1}$
35	2.7	8.43 ± 0.07	2.65 ± 0.02	33.46 ± 0.01	1.75 ± 0.00	19	56.20 ± 1.09	63.51 ± 0.25	12.90 ± 0.48	27.97 ± 13.08	13.0 ± 0.3
	3.4	8.41 ± 0.02	2.63 ± 0.03	33.19 ± 0.09	1.81 ± 0.02	18	55.31 ± 1.58	63.04 ± 0.07	14.32 ± 0.32	38.37 ± 18.19	12.9 ± 0.1
	4.8	8.51 ± 0.03	2.67 ± 0.04	32.62 ± 0.03	1.75 ± 0.01	19	51.55 ± 0.03	62.39 ± 0.22	11.24 ± 0.44	25.26 ± 7.14	12.9 ± 0.9
	7.8	8.60 ± 0.05	2.69 ± 0.01	32.56 ± 0.05	1.85 ± 0.01	18	54.02 ± 4.47	61.09 ± 0.25	9.67 ± 0.46	19.46 ± 7.53	12.7 ± 0.1
45	2.7	5.26 ± 0.02	4.95 ± 0.03	35.30 ± 0.15	1.65 ± 0.00	20	56.23 ± 0.05	63.18 ± 0.52	1461.07 ± 0.16	74.54 ± 10.49	71.9 ± 3.1
	3.4	7.38 ± 0.03	4.55± 0.03	32.37 ± 1.03	1.75 ± 0.03	19	56.06 ± 0.30	62.30 ± 0.19	821.07 ± 39.14	57.63 ± 13.21	33.7 ± 3.7
	4.8	7.42 ± 0.02	4.04± 0.02	33.81 ± 0.47	1.83 ± 0.06	17	51.57 ± 1.02	60.58 ± 0.02	649.90 ± 11.83	80.99 ± 4.68	40.7 ± 1.1
	7.8	7.46 ± 0.01	3.52± 0.01	33.17 ± 0.22	1.77 ± 0.03	18	49.91 ± 0.98	61.14 ± 0.19	457.19 ± 11.43	47.31 ± 11.57	37.5 ± 3.1
55	2.7	8.30 ± 0.01	2.49 ± 0.01	32.03 ± 0.38	1.71 ± 0.03	19	47.54 ± 0.99	60.84 ± 0.46	390.86 ± 12.12	28.00 ± 8.89	36.0 ± 0.8
	3.4	8.29 ± 0.01	2.39 ± 0.01	31.69 ± 0.01	1.85 ± 0.05	17	41.45 ± 1.02	58.42 ± 1.38	137.69 ± 5.44	86.58 ± 3.99	16.0 ± 0.8
	4.8	8.25 ± 0.03	2.28 ± 0.03	31.33 ± 0.71	1.73 ± 0.00	18	38.78 ± 0.78	60.20 ± 0.16	140.44 ± 1.04	24.02 ± 7.63	9.8 ± 0.6
	7.8	8.29 ± 0.01	2.39 ± 0.03	30.21 ± 0.25	1.86 ± 0.02	16	38.55 ± 0.68	59.54 ± 0.03	72.15 ± 3.69	21.25 ± 3.80	13.7 ± 1.1
65	2.7	7.53 ± 0.08	2.84 ± 0.01	36.00 ± 0.21	1.94 ± 0.04	19	59.30 ± 1.44	66.84 ± 0.47	265.36 ± 9.38	10.07 ± 2.78	24.6 ± 0.1
	3.4	7.53 ± 0.05	2.88 ± 0.00	35.94 ± 0.10	2.02 ± 0.02	18	50.74 ± 1.16	67.69 ± 0.17	194.42 ± 6.45	3.25 ± 00	20.7 ± 0.4
	4.8	7.74 ± 0.01	2.56 ± 0.04	35.81 ± 1.16	1.87 ± 0.07	19	51.34 ± 0.11	67.57 ± 0.92	95.25 ± 1.31	25.17 ± 2.47	18.6 ± 1.1
	7.8	7.81 ± 0.02	2.55 ± 0.03	35.17 ± 0.14	2.02 ± 0.06	17	55.33 ± 0.76	66.62 ± 0.38	114.77 ± 0.00	18.23 ± 5.22	32.1 ± 0.7
