# Supplementary figures and images for: Carbon Monoxide Production during Bio-Waste Composting under Different Temperature and Aeration Regimes
Source: Materials (Basel). 2023 Jun 23;16(13):4551. doi: 10.3390/ma16134551 (PMC10342461; doi:10.3390/ma16134551)

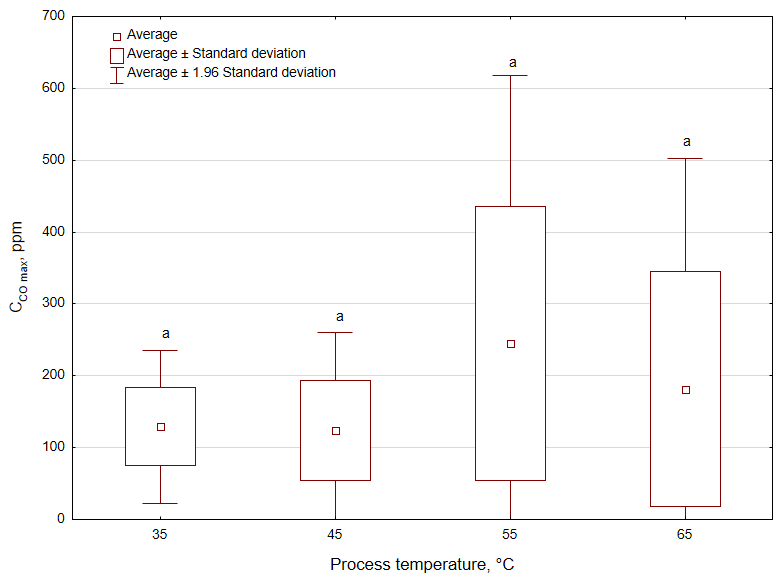

Supplement: Supplementary file 1 [file materials-16-04551-s001.zip › Figure S1.tif]

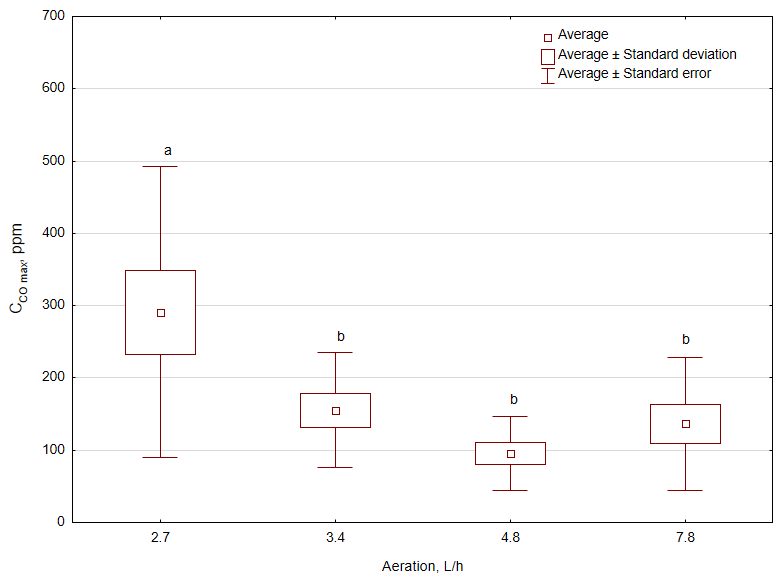

Supplement: Supplementary file 1 [file materials-16-04551-s001.zip › Figure S2.tif]

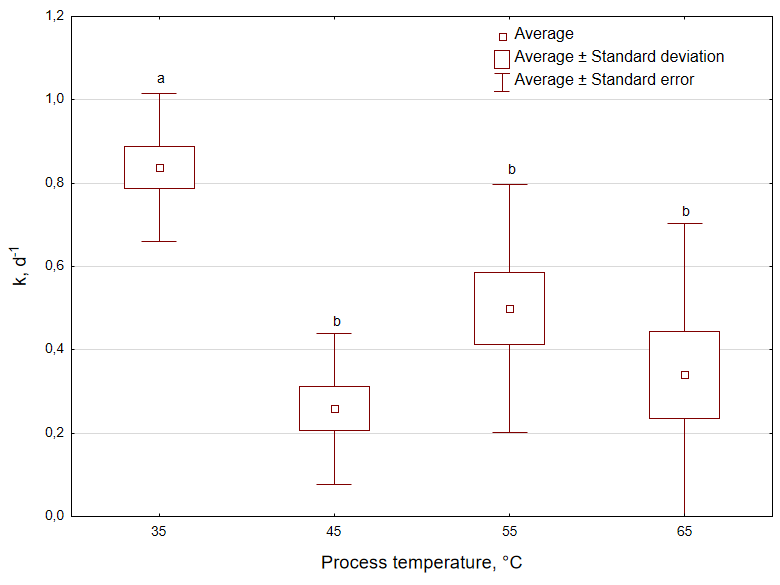

Supplement: Supplementary file 1 [file materials-16-04551-s001.zip › Figure S3.tif]

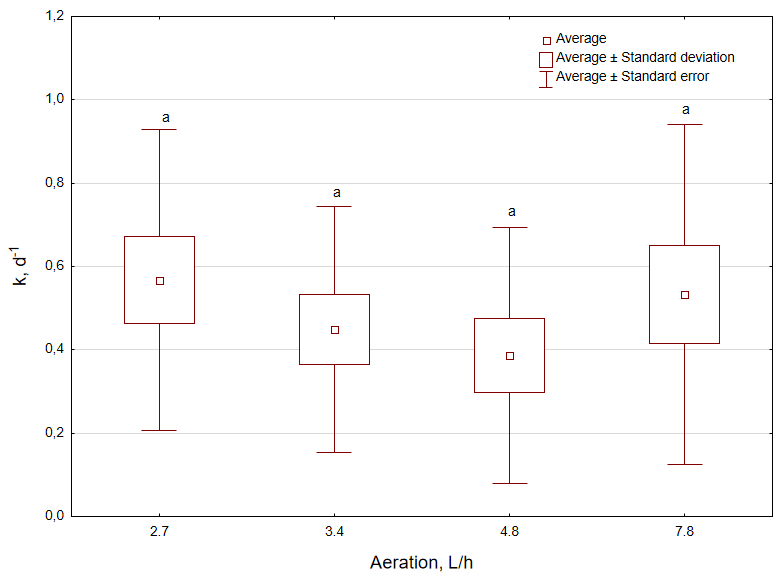

Supplement: Supplementary file 1 [file materials-16-04551-s001.zip › Figure S4.tif]
